# Effect of metformin in addition to an antenatal diet and lifestyle intervention on fetal growth and adiposity: the GRoW randomised trial

**DOI:** 10.1186/s12902-020-00618-0

**Published:** 2020-09-14

**Authors:** Amanda J. Poprzeczny, Jennie Louise, Andrea R. Deussen, Jodie M. Dodd

**Affiliations:** 1 The University of Adelaide, The Robinson Research Institute, and Discipline of Obstetrics and Gynaecology, Adelaide, South Australia Australia; 2 Department of Perinatal Medicine, The Women’s and Children’s Hospital, Women’s and Babies Division, Adelaide, South Australia Australia; 3 The University of Adelaide, Women’s and Children’s Hospital, 72 King William Road, North Adelaide, South Australia 5006 Australia; 4 The University of Adelaide, School of Public Health, Adelaide, South Australia Australia

**Keywords:** Fetal growth, Fetal adiposity, Maternal obesity, Metformin, Antenatal interventions

## Abstract

**Background:**

The infants born to women who are overweight or obese in pregnancy are at an increased risk of being born macrosomic or large for gestational age. Antenatal dietary and lifestyle interventions have been shown to be ineffective at reducing this risk. Our aim was to examine the effects of metformin in addition to a diet and lifestyle intervention on fetal growth and adiposity among women with a BMI above the healthy range.

**Methods:**

Women who had a body mass index ≥25 kg/m^2^ in early pregnancy, and a singleton gestation, were enrolled in the GRoW trial from three public maternity hospitals in metropolitan Adelaide. Women were invited to have a research ultrasounds at 28 and 36 weeks’ gestation at which ultrasound measures of fetal biometry and adiposity were obtained. Fetal biometry z-scores and trajectories were calculated. Measurements and calculations were compared between treatment groups. This secondary analysis was pre-specified.

**Results:**

Ultrasound data from 511 women were included in this analysis. The difference in femur length at 36 weeks’ gestation was (0.07 cm, 95% CI 0.01–0.14 cm, *p* = 0.019) and this was was statistically significant, however the magnitude of effect was small. Differences between treatment groups for all other fetal biometry measures, z-scores, estimated fetal weight, and adiposity measures at 28 and 36 weeks’ gestation were similar.

**Conclusions:**

The addition of metformin to dietary and lifestyle advice in pregnancy for overweight and obese women has no clinically relevant effect on ultrasound measures of fetal biometry or adiposity.

**Trial registration:**

Australian and New Zealand Clinical Trials Registry (ACTRN12612001277831).

## Background

Women commencing pregnancy overweight or obese, defined as a body mass index (BMI) of ≥ 25 kg/m^2^ and ≥ 30 kg/m^2^, respectively,have an increased risk of adverse pregnancy and birth outcomes. These adverse outcomes include gestational diabetes (GDM) [1, 2] and hypertensive disorders, including pre-eclampsia [1, 2], during pregnancy and an increased risk of both induction of labour [1, 3] and birth by caesarean section [1, 4]. In developed countries, overweight and obesity effects approximately 50% of women of reproductive age [5–7].

Infants born to women who are overweight or obese are at increased risk of being born macrosomic or large for gestational age (LGA) [1, 8–10], of birth trauma including shoulder dystocia [10, 11], low Apgar scores, requiring resuscitation at birth [1, 8, 10], hypoglycaemia [12, 13] and requiring neonatal intensive care admission [1, 8, 12]. Longer term, maternal overweight and obesity are independent risk factors for childhood obesity [14–16], which may be partially mediated through an increased risk of infant macrosomia [17].

Antenatal dietary and lifestyle interventions have not been effective in reducing the risk of adverse clinical pregnancy and birth outcomes [18–20]. In particular, such interventions have not reduced infant birthweight or risk of an infant born LGA [18].

Oral metformin, a commonly used insulin sensitising agent, has been evaluated as a strategy to improve pregnancy outcomes for overweight and obese women, given similarities in the metabolic environment between both obesity and GDM. However, studies investigating the effects of antenatal metformin have either not reported growth and development in utero [21], or been limited in their analyses [22], resulting in a dearth of information about the fetal effects of antenatal metformin use.

Our group performed a randomised controlled trial investigating the effects of antenatal metformin therapy as an adjuvant to diet and lifestyle advice for women who were overweight or obese in early pregnancy [23]. We have previously reported that adjuvant antenatal metformin therapy is not associated with any differences in birth weight or risk of infants being born large for gestational age [23]. However, whether adjuvant antenatal metformin therapy has an effect on fetal growth, adiposity, and growth velocity, is not known. The aim of this pre-specified analysis of secondary outcomes was to investigate the effect of this combined antenatal intervention on fetal growth and adiposity.

## Methods

### Participants

The primary findings of the GRoW randomised trial [23] have been published. Women with a singleton, live gestation who were overweight or obese (BMI ≥25.0 kg/m^2^) and between 10 and 20 weeks’ gestation, were eligible to participate. Women with a multiple pregnancy, pre-existing type 1 or type 2 diabetes mellitus, or a contraindication to taking metformin were excluded from the study.

### Randomisation

Potentially eligible women were identified and recruited from three major public maternity hospitals in metropolitan Adelaide – the Women’s and Children’s Hospital, the Lyell McEwin Hospital, and Flinders Medical Centre. Informed consent was obtained. Randomisation used a central computer-based randomisation service, with variable blocks of four. Women were stratified according to parity (0 vs ≥1), BMI at booking visit (25–29.9 kg/m^2^ vs ≥30 kg/m^2^), and collaborating centre. Women, their caregivers, and research staff were blinded to treatment allocation.

### Intervention

Participating women were randomised to either the metformin group or placebo group. Women recruited to the metformin group received a supply of oral metformin tablets (500 mg) and women allocated to the placebo group received a supply of placebo tablets identical in taste and appearance to the metformin tablets. All women were instructed to start taking one tablet per day increasing to a maximum of two tablets twice daily over four weeks as tolerated, and to continue over the course of the pregnancy [23].

All women received a dietary and lifestyle intervention over the course of the study, which we have described in detail previously [23]. This was an individually tailored intervention, and involved three face-to-face sessions over the course of pregnancy (the initial two with a dietitian, and one with a research assistant at 36 weeks’ gestation) and phone calls. Dietary advice was provided, in keeping with Australian dietary standards. This included maintaining a balance of carbohydrates, fat and protein, and reducing energy dense foods high in refined carbohydrates and saturated fats. Women were recommended to consume two servings of fruit, five servings of vegetables, and three servings of dairy each day. Women were encouraged to set and review achievable diet and lifestyle goals and self monitor their progress.

### Ultrasound assessment

An accurate gestational age and estimated date of confinement was calculated for each woman based on early pregnancy ultrasound or last menstrual period. Women underwent a routine fetal anomaly scan at 18–20 weeks’ gestation, in keeping with South Australian Perinatal Practice Guidelines [24]. Participating women gave permission for fetal biometry results from this scan to be made available to the researchers. All women were invited to attend for a research ultrasound at 28 (range 26^+ 0^ to 29^+ 6^) and 36 (range 34^+ 0^ to 37^+ 6^) weeks’ gestation. A medical practitioner with specialist or subspecialist training in obstetric ultrasound performed all research ultrasounds, and was blinded to the woman’s allocated treatment group.

#### Fetal biometry measures

Fetal biometry measures collected from the routine fetal anomaly scan at 18–20 weeks’ gestation included standard measurements of head circumference (HC), biparietal diameter (BPD), abdominal circumference (AC) and femur length (FL). At the research ultrasounds, measurements of standard fetal biometry (HC, BPD, AC, and FL) were obtained in accordance with national and international standards of practice [25]. Biometry measures obtained from the research ultrasounds were converted into z scores to allow for variation in gestational age and fetal sex, using recognised Australian population standards [25, 26]. Estimated fetal weight (EFW) was calculated using the Hadlock C formula [27].

#### Fetal growth velocities

Fetal growth velocities are presented as the difference between 28 week measures and 36 week measures, calculated as total change / actual number of days between measurements. Velocity z-scores were likewise calculated using recognised Australian population standards where available [28].

#### Fetal adiposity measures

Fetal subcutaneous tissue fat thickness measurements were obtained at both research ultrasounds. These measurements included mid thigh lean mass (MTLM), mid thigh fat mass (MTFM), abdominal fat mass (AFM), and subscapular fat mass (SSFM), and were obtained by methods described previously [29–34]. Mid thigh total, lean and fat mass were obtained by taking a longitudinal view of the femur, then rotating the transducer through 90 degrees to obtain a cross-sectional view of the mid-thigh [30, 31]. MTFM was measured by taking the total cross-sectional limb area (MTTM) and subtracting MTLM (consisting of the central lean area comprising muscle and bone). Fetal AFM was measured at the level of the abdominal circumference, between fetal mid-axillary lines and anterior to the margins of the ribs [29, 30]. This was measured in millimetres and using magnification. The SSFM was obtained by a sagittal view of the fetal trunk, to view the entire longitudinal section of the scapula. The subcutaneous fat tissue measurement was taken at the level of the end of the scapula [30]. We have previously shown good inter-observer variability for these measurements in a similar cohort of women who were overweight or obese in early pregnancy [33].

### Statistical analysis

Baseline characteristics of women included in the analysis were compared descriptively between treatment groups. Continuous variables were reported as means and standard deviations, or as medians and interquartile ranges if not normally distributed. Categorical variables were reported as frequencies and percentages. Analyses were performed on available data on an intention-to-treat basis, with women analysed according to the treatment group into which they were randomised.

Outcomes measured at multiple time points were analysed using linear regression models, with Generalised Estimating Equations to account for correlation due to repeated measures, and a time-by-treatment interaction term to test for differences in treatment effect between time points. Estimates are reported as differences in means (metformin group – placebo group) and 95% confidence intervals for each time point separately, regardless of the significance of the interaction term. Growth velocity outcomes were analysed using linear regression models, with estimates again reported as differences in means (metformin group – placebo group) and 95% confidence intervals. Both unadjusted and adjusted analyses were performed, with adjusted analyses including stratification variables (study centre, BMI category and parity), maternal age at trial entry, smoking status, and Socio-Economic Indexes for Areas Index of Relative Socio-Economic Disadvantage (SEIFA IRSD) quintile as covariates. All analyses were performed using SAS v9.4 (SAS Institute, Cary, NC).

## Results

### Participant characteristics

**Fig. 1:**
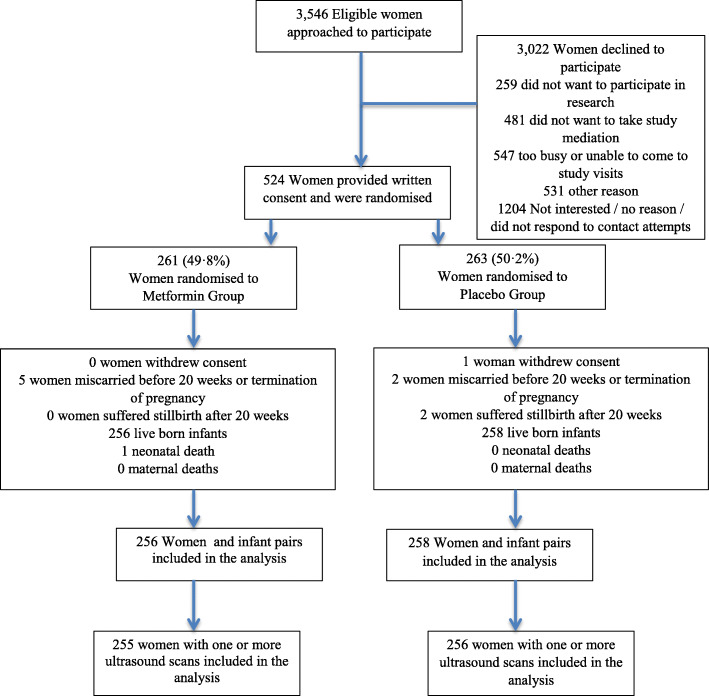
Flow of women eligible for inclusion in the analysis of ultrasound measurements of fetal growth and adiposity in the GRoW randomised trial

A total of 524 women were recruited and randomised in the GRoW randomised trial, with 261 (49.8%) women randomised to the metformin group, and 263 (50.2%) women randomised to the placebo group. Overall, 10 women withdrew, underwent a termination of pregnancy, or suffered a miscarriage before 20 weeks’ gestation, resulting in a total of 256 women and their infants in the metformin group and 258 women and their infants in the placebo group who were included in primary analyses (Fig. 1). The present study includes a total of 511 women who attended for one or more research ultrasounds, 255 women from the metformin group and 256 women from the placebo group (Fig. 1).

Baseline demographic characteristics of participating women are shown in Table 1, and were comparable between treatment groups. The median gestational age at trial entry was 16.29 weeks (Interquartile Range (IQR) 14.43–18.00 weeks). The median BMI of the cohort was 32.30 kg/m^2^ (IQR 28.90–37.20 kg/m^2^), with most women in their second or subsequent pregnancy, non-smokers, and 62.63% from the highest two quintiles of social disadvantage [35]. These characteristics are similar to the full randomised cohort [23].

**Table 1:** Baseline characteristics of women recruited to the GRoW randomised trial who attended for one or more research ultrasounds

Characteristic	Metformin Group *N* = 255	Placebo Group *N* = 256	Total *N* = 511
Maternal age (years; mean (SD))	29.88 (5.55)	30.15 (5.38)	30.02 (5.46)
Gestational age at trial entry (weeks) Median (IQR)	16.29 (14.43, 18.00)	16.21 (14.57, 18.07)	16.29 (14.43, 18.00)
BMI (kg/m^2^) Median (IQR)	32.40 (28.70, 37.57)	32.05 (29.10, 36.80)	32.30 (28.90, 37.20)
BMI category (kg/m^2^) N (%):			
BMI 25.0–29.9	83 (32.55)	83 (32.42)	166 (32.49)
BMI 30.0–34.9	74 (29.02)	83 (32.42)	157 (30.72)
BMI 35.0–39.9	58 (22.75)	48 (18.75)	106 (20.74)
BMI **≥** 40.0	40 (15.69)	42 (16.41)	82 (16.05)
Nulliparity N (%)	88 (34.51)	91 (35.55)	179 (35.03)
Smoker N (%)	24 (9.41)	43 (16.80)	67 (13.11)
Height at trial entry (cm) Mean (SD)	165.24 (6.76)	164.89 (6.78)	165.07 (6.76)
Weight at trial entry (kg) Mean (SD)	92.86 (19.79)	91.87 (19.76)	92.36 (19.76)
Ethnicity N(%)			
Caucasian	209 (81.96)	219 (85.55)	428 (83.76)
Asian	5 (1.96)	7 (2.73)	12 (2.35)
Aboriginal/Torres Strait Islander	6 (2.35)	7 (2.73)	13 (2.54)
African	6 (2.35)	0 (0.00)	6 (1.17)
Other/unknown	29 (11.38)	23 (8.98)	52 (10.18)
SEIFA IRSD Quintile N(%)			
Quintile 1	75 (29.41)	93 (36.33)	168 (32.88)
Quintile 2	78 (30.59)	74 (28.91)	152 (29.75)
Quintile 3	31 (12.16)	30 (11.72)	61 (11.94)
Quintile 4	52 (20.39)	43 (16.80)	95 (18.59)
Quintile 5	19 (7.45)	16 (6.25)	35 (6.85)

### Fetal biometry measures

There were no statistically significant differences in fetal biometry measures of BPD, HC or AC at any of the time points assessed (20 weeks’, 28 weeks’, and 36 weeks’ gestation; Table 2). Measurements of FL were not statistically significantly different at 20 or 28 weeks’ gestation (Table 2). Average femur length was statistically significantly greater in the metformin group at 36 weeks’ gestation, however the magnitude of the difference was small – only 0.07 (95% CI: 0.01, 0.14) cm. There were no statistically significant differences seen in calculated EFW at 28 or 36 weeks’ gestation, by treatment group (Table 2). The estimates of effect size for fetal biometry measures were all less than 0.50 cm and crossed zero (Table 2), suggesting that there were little to no absolute differences in average fetal biometry measures between the metformin and placebo groups, and that the true effect of treatment is not clinically relevant. Similarly, the estimated effect size on estimated fetal weight was only 17.61 (95% CI -57.67, 92.88) gm (Table 2), again suggesting a clinically relevant effect is unlikely.

**Table 2:** Effect of adjuvant metformin on ultrasound measures of fetal biometry over pregnancy

Outcome	Time point	Metformin group Mean (SD)	Control group Mean (SD)	Unadjusted treatment effect (95% CI)	Unadjusted *P*-value	Adjusted treatment effect (95% CI)	Adjusted P-value
Biparietal diameter (cm)					0.138*		0.102*
	20 weeks	4.66 (0.34)	4.69 (0.35)		0.328		0.232
	28 weeks	7.13 (0.50)	7.15 (0.41)	−0.02 (− 0.10, 0.06)	0.645	− 0.02 (− 0.10, 0.07)	0.671
	36 weeks	8.94 (0.42)	8.88 (0.39)	0.06 (−0.01, 0.14)	0.110	0.07 (− 0.01, 0.14)	0.076
Head circumference (cm)					0.299*		0.200*
	20 weeks	17.38 (1.20)	17.45 (1.36)	−0.07 (−0.29, 0.16)	0.551	−0.09 (− 0.31, 0.13)	0.431
	28 weeks	26.46 (1.55)	26.43 (1.26)	0.04 (−0.22, 0.30)	0.744	0.05 (−0.22, 0.31)	0.727
	36 weeks	32.36 (1.23)	32.20 (1.21)	0.17 (−0.05, 0.40)	0.134	0.19 (−0.04, 0.42)	0.100
Femur length (cm)					0.018*		0.012*
	20 weeks	3.26 (0.30)	3.28 (0.30)	−0.03 (−0.08, 0.02)	0.267	−0.04 (− 0.09, 0.01)	0.127
	28 weeks	5.29 (0.37)	5.29 (0.30)	0.01 (−0.06, 0.07)	0.841	0.00 (−0.06, 0.07)	0.909
	36 weeks	6.92 (0.32)	6.85 (0.33)	0.08 (0.01, 0.14)	0.015	0.07 (0.01, 0.14)	0.019
Abdominal circumference (cm)					0.992*		0.959
	20 weeks	15.56 (1.37)	15.65 (1.29)	−0.09 (−0.32, 0.14)	0.457	−0.10 (− 0.32, 0.12)	0.368
	28 weeks	24.62 (1.75)	24.70 (1.56)	−0.07 (− 0.37, 0.24)	0.669	− 0.05 (− 0.36, 0.26)	0.747
	36 weeks	32.82 (1.87)	32.92 (1.99)	−0.07 (− 0.43, 0.29)	0.690	− 0.07 (− 0.43, 0.29)	0.707
Estimated fetal weight (g)					0.720*		0.756*
	28 weeks	1272.36 (248.33)	1268.81 (201.23)	5.05 (−36.72, 46.82)	0.813	5.40 (−36.93, 47.74)	0.803
	36 weeks	2932.32 (399.97)	2914.27 (417.68)	18.98 (−56.99, 94.95)	0.624	17.61 (−57.67, 92.88)	0.647

*denotes *p* value for test of interaction between treatment and time, i.e. whether treatment effect varies over time

In keeping with the above findings, there were no statistically significant differences in the fetal biometry z-scores of BPD, HC, FL or AC between the treatment groups, at any of the time points assessed (supplementary Table 1). There were no statistically significant differences in calculated estimated fetal weight z-score at 28 or 36 weeks’ gestation, by treatment group (supplementary Table 1). There was no evidence that the treatment effect differed between time points. All fetal biometry measure z-scores were positive in both groups, at all time points, suggesting the fetuses of women in our study were larger on average than the reference population used [26]. With regards to fetal biometry velocities and estimated fetal weight velocity, there were no statistically significant differences by treatment group, either in the raw velocity measurements or velocity for z-score (supplementary Table 2).

### Fetal adiposity measures

There was no statistically significant treatment effect on any of the measures of fetal adiposity, at either 28 or 36 weeks’ gestation (Table 3). Estimates of effect size for all adiposity measures were close to zero, and the range of the 95% confidence intervals were small, suggesting a clinically meaningful effect is unlikely.

**Table 3:** Effect of adjuvant metformin treatment on ultrasound fetal adiposity measures over pregnancy

Outcome	Time point	Metformin group Mean (SD)	Control group Mean (SD)	Unadjusted treatment effect (95% CI)	Unadjusted *P*-value	Adjusted treatment effect (95% CI)	Adjusted P-value
Mid-thigh fat mass (cm^2^)					0.845*		0.843*
	28 weeks	4.24 (1.03)	4.21 (1.09)	0.04 (−0.27, 0.35)	0.805	0.02 (− 0.29, 0.33)	0.902
	36 weeks	9.87 (2.79)	9.95 (2.47)	−0.05 (− 0.95, 0.84)	0.908	− 0.07 (− 0.94, 0.80)	0.870
Abdominal fat mass (mm)					0.144*		0.199*
	28 weeks	3.60 (1.17)	3.60 (1.08)	0.01 (−0.31, 0.34)	0.935	0.06 (−0.27, 0.39)	0.718
	36 weeks	6.42 (1.41)	5.98 (1.51)	0.42 (−0.05, 0.88)	0.077	0.42 (−0.05, 0.89)	0.079
Subscapular fat mass (mm)					0.919*		0.655*
	28 weeks	3.22 (0.80)	3.20 (0.88)	0.02 (−0.21, 0.25)	0.863	0.03 (−0.19, 0.25)	0.760
	36 weeks	4.84 (1.44)	4.85 (1.48)	−0.00 (− 0.42, 0.41)	0.985	− 0.07 (− 0.48, 0.34)	0.740

*denotes *p* value for test of interaction between treatment and time, i.e. whether treatment effect varies over time

## Discussion

### Main findings

Our findings demonstrate that, among pregnant women who are overweight or obese, antenatal treatment with oral metformin as an adjunct to dietary and lifestyle advice did not appreciably impact measures of fetal biometry or adiposity, or fetal biometry growth velocities over the third trimester of pregnancy.

### Interpretation

Metformin has been used increasingly in the treatment of GDM. The Metformin in Gestational Diabetes (MiG) Trial confirmed the safety and efficacy of metformin use in women with GDM [21]. Oral metformin was not associated with an increased rate of a composite neonatal adverse outcome (made up of neonatal hypoglycaemia, respiratory distress, need for phototherapy, birth trauma, 5-min Apgar < 7, or preterm birth) [21], confirming its safety in pregnancy. Rowan et al also found no significant differences between metformin and insulin with regards to neonatal biometry, circumferences, or skin-fold thickness measurements [21].

Subsequent childhood follow up at two years of age, however, revealed selected differences with children exposed to metformin in pregnancy having statistically significantly greater upper arm circumference and subscapular and biceps skinfold thicknesses, and greater fat free mass measurements, in comparison with children exposed to insulin antenatally [36]. It was hypothesised that antenatal metformin caused fat to be stored in subcutaneous sites, resulting in less ectopic or visceral fat [36]. The MiG trial did not report fetal measures of growth and adiposity, however, and our findings refute this hypothesis, as adjuvant antenatal metformin started in the second trimester among women who were overweight or obese in early pregnancy was not associated with any differences in subcutaneous tissue fat measures results in our cohort. Women recruited to the MiG study had an average early pregnancy BMI of 32 kg/m^2^ [21], similar to women in the GRoW randomised cohort [23]. In contrast to the MiG study, however, women recruited to the GRoW randomised trial commenced treatment much earlier in pregnancy, with an average gestational age at trial entry of 16 weeks [23].

Our findings are in contrast to those reported in the PregMet2 randomised trial [37], investigating the effect of adjuvant antenatal metformin treatment among women with polycystic ovary syndrome (PCOS), starting in early pregnancy. While the primary outcome of this study was late miscarriage and preterm birth, fetal and neonatal biometry measures were performed in the third trimester and after birth as a secondary outcome [37]. This group found that the fetuses of women exposed to antenatal metformin had statistically significantly larger BPD measurements at 32 weeks’ gestation, and greater HC measurements at birth [22]. However, the magnitude of these differences was small – on average, less than one centimetre – and the authors concluded that these differences, while statistically significant, were not likely to be clinically relevant [37]. The population of women recruited to the PregMet2 randomised trial are different to those women recruited to the GRoW randomised trial, in that they all had a diagnosis of PCOS and, while the average BMI at trial entry was 29 kg/m^2^, this is lower than the average BMI at trial entry in the GRoW randomised trial, which was 32 kg/m^2^ [23]. This study was also smaller than the GRoW randomised trial, recruiting approximately half the number of the GRoW Trial.

Our work builds on that of two other published randomised trials investigating the effect of antenatal metformin among women who were obese in early pregnancy [38, 39]. The Effect of Metformin on Maternal and Fetal Outcomes in Obese Pregnant Women (EMPOWaR) randomised trial [38] randomised women with a BMI ≥ 30 kg/m^2^ between 12 and 16 weeks’ gestation to receive either metformin up to 2.5 g per day or placebo. They found no significant difference in mean birthweight or birthweight z-score [38], similar to the primary outcome of the GRoW trial [23]. The second trial, Metformin in Obese Nondiabetic Pregnant Women (MOP) trial [39], randomised women with a BMI ≥ 35 kg/m^2^ between 12 and 18 weeks’ gestation, to metformin up to 3 g daily or placebo. Similarly, they found no statistically significant difference in birthweight z-score or incidence of neonatal LGA [39].

Our group has previously shown, in the LIMIT randomised trial [40], that an antenatal diet and lifestyle intervention in women who were overweight or obese was associated with greater fetal mean mid-thigh fat mass, and a significantly slower rate of subscapular adipose tissue deposition, with no difference in lean thigh mass or abdominal fat mass [33]. All women randomised to the GRoW trial were exposed to the same dietary and lifestyle intervention as was provided in the LIMIT randomised trial [40]. Of interest, results of fetal biometry z scores, estimated fetal weights and subcutaneous fat measures obtained from fetuses of women randomised to the GRoW trial placebo group were similar to those found in fetuses randomised to the diet and lifestyle group of the LIMIT randomised trial [40], providing further support for the robustness of our measurements in these very similar populations. While we have hypothesised previously that an antenatal diet and lifestyle intervention for women who were overweight or obese in early pregnancy may be associated with a more favourable fetal fat phenotype [33], our current findings do not suggest that there is any further effect on fetal adiposity with the addition of metformin to the aforementioned antenatal diet and lifestyle intervention. It is possible that the pathways involved in fetal fat deposition have been “saturated” by the effect of the diet and lifestyle intervention and cannot be further affected by the addition of metformin.

Women recruited to the GRoW randomised trial were, on average, 16 weeks’ gestation (Table 1). There is increasing evidence that the preconceptual period is of vital importance to healthy growth and development [41, 42]. Sovio and colleagues [43] demonstrated that the fetuses of women who were obese were more likely to have an abdominal circumference measurement greater than the 90th percentile which was already evident at 20 weeks’ gestation. It is possible that interventions need to be commenced prior to conception, to have an appreciable effect on fetal growth and adiposity.

## Conclusions

Based on our study results, there is no evidence that the addition of metformin to dietary and lifestyle advice in pregnancy for overweight and obese women has a clinically relevant effect on ultrasound measures of fetal biometry or adiposity.

## Supplementary information

**Additional file 1: Supplementary Table 1.** Effect of adjuvant antenatal metformin treatment on fetal biometry z-scores across pregnancy. **Supplementary Table 2.** Effect of adjuvant antenatal metformin treatment on fetal biometry velocities across pregnancy.

## Data Availability

The data that supports the findings of this study are available from the authors upon reasonable request and cannot be made publicly available, due to the nature of the data and the ethics approval obtained.

## Abbreviations

GRoW randomised trial: metformin and dietary advice to improve insulin sensitivity and promote gestational restriction of weight among pregnant women who are overweight or obese randomised trial
MoP study: metformin in obese non-diabetes pregnant women randomised trial
MiG study: metformin in gestational diabetes trial
LIMIT randomised trial: limiting weight gain in overweight and obese women during pregnancy to improve health outcomes: a randomised trial
BMI: body mass index
GDM: gestational diabetes
LGA: large for gestational age
HC: head circumference
BPD: biparietal diameter
AC: abdominal circumference
FL: femur length
EFW: estimated fetal weight
MTLM: mid-thigh lean mass
MTFM: mid-thigh fat mass
AFM: abdominal fat mass
SSFM: subscapular fat mass
SEIFA IRSD: socio-economic indexes for areas index of relative socio-economic disadvantage
IQR: interquartile range
