# Supplementary material for: Effect of metformin in addition to an antenatal diet and lifestyle intervention on fetal growth and adiposity: the GRoW randomised trial
Source: BMC Endocr Disord. 2020 Sep 14;20:139. doi: 10.1186/s12902-020-00618-0 (PMC7488668; doi:10.1186/s12902-020-00618-0)
Supplement: Supplementary file 1 — Additional file 1: Supplementary Table 1. Effect of adjuvant antenatal metformin treatment on fetal biometry z-scores across pregnancy. Supplementary Table 2. Effect of adjuvant antenatal metformin treatment on fetal biometry velocities across pregnancy. [file 12902_2020_618_MOESM1_ESM.docx]

**Supplementary table 1**. Effect of adjuvant antenatal metformin treatment on fetal biometry z-scores across pregnancy.

*denotes p value for test of interaction between treatment and time, i.e. whether treatment effect varies over time.

| Outcome (z score) | Time point | Metformin group  Mean (SD) | Control group  Mean (SD) | Unadjusted treatment effect (95% CI) | Unadjusted P-value | Adjusted treatment effect (95% CI) | Adjusted P-value |
| --- | --- | --- | --- | --- | --- | --- | --- |
| Biparietal diameter |  |  |  |  | 0.138* |  | 0.102* |
|  | 28 weeks | 0.41 (1.54) | 0.56 (1.47) | -0.14 (-0.41 , 0.14) | 0.333 | -0.13 (-0.41, 0.14) | 0.335 |
|  | 36 weeks | 0.18 (1.23) | 0.10 (1.14) | 0.04 (-0.18, 0.26) | 0.697 | 0.06 (-0.16, 0.28) | 0.564 |
| Head circumference |  |  |  |  | 0.788* |  | 0.708* |
|  | 28 weeks | 0.82 (1.02) | 0.83 (0.88) | 0.02 (-0.15 , 0.20) | 0.817 | 0.02 (-0.15, 0.20) | 0.801 |
|  | 36 weeks | 0.85 (0.89) | 0.79 (0.86) | 0.05 (-0.12, 0.21) | 0.583 | 0.06 (-0.11, 0.22) | 0.493 |
| Femur length |  |  |  |  | 0.036* |  | 0.031* |
|  | 28 weeks | 0.30 (0.89) | 0.37 (0.95) | -0.06 (-0.23 , 0.11) | 0.496 | -0.09 (-0.26, 0.08) | 0.316 |
|  | 36 weeks | 0.36 (1.03) | 0.21 (1.03) | 0.14 (-0.05, 0.32) | 0.161 | 0.12 (-0.07, 0.30) | 0.230 |
| Abdominal circumference |  |  |  |  | 0.743* |  | 0.696* |
|  | 28 weeks | 0.61 (0.92) | 0.75 (0.98) | -0.13 (-0.31 , 0.04) | 0.134 | -0.12 (-0.30, 0.05) | 0.165 |
|  | 36 weeks | 0.56 (1.06) | 0.73 (1.01) | -0.16 (-0.35, 0.03) | 0.098 | -0.16 (-0.35, 0.03) | 0.104 |
| Estimated fetal weight |  |  |  |  | 0.890* |  | 0.901* |
|  | 28 weeks | 0.32 (0.85) | 0.40 (0.89) | -0.07 (-0.23 , 0.09) | 0.399 | -0.07 (-0.23, 0.09) | 0.398 |
|  | 36 weeks | 0.42 (0.97) | 0.48 (0.93) | -0.06 (-0.23, 0.12) | 0.513 | -0.06 (-0.23, 0.11) | 0.500 |

**Supplementary table 2.** Effect of adjuvant antenatal metformin treatment on fetal biometry velocities across pregnancy.

| Outcome | Metformin group  Mean (SD) | Control group  Mean (SD) | Unadjusted treatment effect (95% CI) | Unadjusted P-value | Adjusted treatment effect (95% CI) | Adjusted P-value |
| --- | --- | --- | --- | --- | --- | --- |
| BPD (cm/wk) | 0.22 (0.04) | 0.22 (0.04) | 0.000 (-0.008, 0.009) | 0.967 | -0.003 (-0.011, 0.006) | 0.555 |
| HC (cm/wk) | 0.11 (0.02) | 0.11 (0.02) | -0.002 (-0.006, 0.002) | 0.398 | -0.003 (-0.007, 0.001) | 0.150 |
| FL (cm/wk) | 0.03 (0.00) | 0.03 (0.01) | 0.000 (-0.001, 0.001) | 0.403 | 0.000 (-0.001, 0.001) | 0.491 |
| Abdominal area (AA) (cm^2^/wk) | 0.67 (0.14) | 0.70 (0.14) | -0.021 (-0.048, 0.005) | 0.118 | -0.021 (-0.049, 0.006) | 0.127 |
| EFW (g/wk) | 29.93 (5.20) | 30.32 (5.32) | -0.384 (-1.412, 0.645) | 0.465 | -0.405 (-1.460, 0.650) | 0.452 |
| BPD z score | 0.80 (1.08) | 0.69 (1.08) | 0.104 (-0.106, 0.314) | 0.331 | 0.034 (-0.175, 0.242) | 0.751 |
| AA z score | 0.47 (0.94) | 0.60 (0.97) | -0.132 (-0.317, 0.054) | 0.164 | -0.133 (-0.322, 0.057) | 0.170 |
| FL z score | 0.70 (0.98) | 0.56 (1.12) | 0.144 (-0.061, 0.349) | 0.168 | 0.128 (-0.081, 0.337) | 0.231 |
| EFW z score | 0.69 (1.10) | 0.77 (1.14) | -0.080 (-0.298, 0.139) | 0.476 | -0.084 (-0.309, 0.140) | 0.462 |
